# *In silico *discovery of transcription regulatory elements in *Plasmodium falciparum*

**DOI:** 10.1186/1471-2164-9-70

**Published:** 2008-02-07

**Authors:** Jason A Young, Jeffery R Johnson, Chris Benner, S Frank Yan, Kaisheng Chen, Karine G Le Roch, Yingyao Zhou, Elizabeth A Winzeler

**Affiliations:** 1 Department of Cell Biology, ICND 202, The Scripps Research Institute, 10550 North Torrey Pines Road, La Jolla, CA 92037, USA; 2 Department of Cellular and Molecular Medicine, University of California - San Diego, La Jolla, CA, USA; 3 Department of Bioengineering, University of California - San Diego, La Jolla, CA, USA; 4 Genomics Institute of the Novartis Research Foundation, San Diego, CA, USA; 5 Department of Cell Biology and Neuroscience, University of California - Riverside, Riverside, CA, USA

## Abstract

**Background:**

With the sequence of the *Plasmodium falciparum *genome and several global mRNA and protein life cycle expression profiling projects now completed, elucidating the underlying networks of transcriptional control important for the progression of the parasite life cycle is highly pertinent to the development of new anti-malarials. To date, relatively little is known regarding the specific mechanisms the parasite employs to regulate gene expression at the mRNA level, with studies of the *P. falciparum *genome sequence having revealed few *cis*-regulatory elements and associated transcription factors. Although it is possible the parasite may evoke mechanisms of transcriptional control drastically different from those used by other eukaryotic organisms, the extreme AT-rich nature of *P. falciparum *intergenic regions (~90% AT) presents significant challenges to *in silico cis*-regulatory element discovery.

**Results:**

We have developed an algorithm called Gene Enrichment Motif Searching (GEMS) that uses a hypergeometric-based scoring function and a position-weight matrix optimization routine to identify with high-confidence regulatory elements in the nucleotide-biased and repeat sequence-rich *P. falciparum *genome. When applied to promoter regions of genes contained within 21 co-expression gene clusters generated from *P. falciparum *life cycle microarray data using the semi-supervised clustering algorithm Ontology-based Pattern Identification, GEMS identified 34 putative *cis*-regulatory elements associated with a variety of parasite processes including sexual development, cell invasion, antigenic variation and protein biosynthesis. Among these candidates were novel motifs, as well as many of the elements for which biological experimental evidence already exists in the *Plasmodium *literature. To provide evidence for the biological relevance of a cell invasion-related element predicted by GEMS, reporter gene and electrophoretic mobility shift assays were conducted.

**Conclusion:**

This GEMS analysis demonstrates that *in silico *regulatory element discovery can be successfully applied to challenging repeat-sequence-rich, base-biased genomes such as that of *P. falciparum*. The fact that regulatory elements were predicted from a diverse range of functional gene clusters supports the hypothesis that *cis*-regulatory elements play a role in the transcriptional control of many *P. falciparum *biological processes. The putative regulatory elements described represent promising candidates for future biological investigation into the underlying transcriptional control mechanisms of gene regulation in malaria parasites.

## Background

While intense research efforts have focused on understanding how gene expression is regulated in model organisms, there are thousands of species important to human health, the environment, and global economies whose transcriptional control mechanisms are not well represented by current biological models. One such species is the apicomplexan parasite responsible for the most lethal form of malaria in humans,*Plasmodium falciparum*. When the *P. falciparum *genome sequence was published in 2002, it was revealed that the nucleotide composition was unusually AT-rich (~80% AT on average, ~90% AT in intergenic regions) with approximately 60% of the predicted genes possessing no known function [1]. Furthermore, initial analyses of the genome using BLAST and profile-Hidden Markov Model searches suggested an apparent dearth of transcription factors [1-3] leading to much speculation that the parasite relied primarily on post-transcriptional regulatory mechanisms for control of its gene expression.

However, over the past 15 years, several investigators have identified on a gene-by-gene basis using traditional experimental approaches regions of gene promoters, and in some cases specific sequence elements, that are important for proper gene expression [4-12]. Additionally, microarray expression data have shown that for the majority of genes, transcript levels vary significantly between different stages of the parasite life cycle [13,14] and the recent applications of more sensitive bioinformatic methods such as two-dimensional hydrophobic cluster analysis coupled with profile-based search methods have identified additional components of the core transcription machinery [15]. Thus, although post-transcriptional mechanisms such as anti-sense transcription [16-19], selective repression of transcript translation [20-22], or epigenetic mechanisms [23] are likely to play crucial roles in the regulation of parasite gene expression, a central role for transcriptional regulation in regulating proper gene expression in *P. falciparum *cannot yet be ruled out.

With the recent emergence of genomic sequences and associated transcriptome datasets for many species, *in silico *methods of *cis*-regulatory element discovery offer much promise towards rapidly elucidating mechanisms of transcriptional control. This is especially true in non-model organisms such as *P. falciparum *where traditional genetic and biochemical experimental methods have been slow to yield insights. Some examples of the most commonly used approaches include MEME [24], AlignACE [25], MDScan [26], and Weeder [27] (for a comprehensive review see [28]). Most of these methods utilize some type of statistical background-modeling approach to identify putative transcription factor binding sites as sequence motifs that occur in the promoter regions of co-expressed genes in greater frequency than would be expected if a random set of promoter regions were considered (i.e. the background). Although successful when applied to organisms possessing well-annotated genomes of AT contents between 40% and 70% [29], we have found that these methods tend to produce an undesirably high number of false positive regulatory elements when applied to AT-rich *P. falciparum *promoter sequences. Thus, to overcome the challenges posed to *in silico cis*-regulatory element discovery by the AT-rich *P. falciparum *genome, we have developed an algorithm called Gene Enrichment Motif Searching (GEMS).

When applied to the *P. falciparum *genome, GEMS was able to identify putative *cis*-regulatory elements in the repeat-sequence-rich base-biased genome by: 1) using a hypergeometric-based scoring function to analyze empirical sequence data without the use of repeat masking; 2) eliminating the guesswork of mismatch and similarity threshold selection by using an exhaustive parameter optimization routine to determine the best representation of putative *cis*-regulatory elements as position-weight matrices (PWMs).

When applied to promoter regions of genes contained within 21 functionally-enriched co-expression gene clusters generated from *P. falciparum *life cycle microarray expression data using the semi-supervised clustering algorithm Ontology-based Pattern Identification (OPI) [30], GEMS identified 34 high-confidence putative *cis*-regulatory elements including many of *cis*-regulatory elements previously described in *P. falciparum *literature. These 34 motif candidates were found in the promoter regions of genes associated with a wide variety of parasite processes including sexual development, antigenic variation, cell invasion, sporozoite development, ribosome function and DNA replication, thus supporting the hypothesis that *cis*-regulatory elements play an important role in the transcriptional control of a diverse array of *P. falciparum *biological processes. Additional support for the biological relevance of these motifs was given by comparative genomic analyses of orthologous promoter sequences from rodent malaria species and detection of element positional enrichment relative to gene start codons. Furthermore, the function of a regulatory element associated with cell invasion genes described herein was characterized using reporter gene and electrophoretic mobility shift assays (EMSAs). Collectively, these results provide much needed robust starting points for the future biological characterization of *cis*-regulatory elements in *P. falciparum *and demonstrate in general that *in silico *approaches to understanding transcriptional regulation mechanisms can be successfully used to predict regulatory elements in non-model organisms possessing unusual genome characteristics.

## Results and discussion

### Gene Cluster Generation using Ontology-based Pattern Identification

Central to *in silico *methods of *cis*-regulatory element discovery is the assumption that co-transcribed genes involved in similar biological processes are also likely to be controlled by common regulatory mechanisms. Thus, if one is interested in finding potential promoter regulatory elements important to these transcriptional control mechanisms, one should search the upstream regions of co-expressed or functionally-related genes for conserved sequence motifs common to these genes but not abundant in the remainder of the genome. Every GEMS analysis therefore begins with a cluster of genes hypothesized to share upstream *cis*-regulatory elements. These gene clusters, defined as positive gene sets, often times are generated simply by using criteria such as common function or membership within a sub-cellular complex. Although successful in some instances [9], this approach by itself is not well suited for analysis of *P. falciparum *as approximately 60% of genes in the genome have no known function [1]. Thus, use of annotation alone in cluster formation results in positive sets that are relatively incomplete for many parasite-specific processes and sub-cellular structures. Furthermore, and at a more fundamental level, common function is not necessarily the best indicator for whether two genes will share a common regulatory element as many times two genes of similar function will exhibit very different expression patterns throughout the life cycle as is the case with many kinases and proteases.

Co-expression at the mRNA level represents a better basis for identifying putative regulatory elements because steady state mRNA levels are a more direct estimation of the effects *cis*-acting regulatory elements have on transcriptional activity. With this in mind, we chose to use the semi-supervised clustering algorithm OPI that utilizes the information currently available on *P. falciparum *gene function from sources such as the Gene Ontology (GO) database [31] to guide the clustering of genes based on microarray-derived life cycle mRNA expression patterns [30]. This resulted in the generation of clusters that contain genes that are both functionally related and highly co-expressed. From the 381 gene clusters derived from 38 *P. falciparum *asexual and sexual stage expression microarray data sets obtained in our most recent OPI analysis of 3059 genes differentially expressed (fold change > 1.5, one-way pANOVA < 0.2) [32], we selected 20 representing a broad range of biological functions and expression patterns as the basis for subsequent GEMS analysis. Additionally, we also assembled a sporozoite-specific set of genes to seed a new OPI cluster (GO:GNF0006) recognizing that mosquito stage gene functions are not well represented in the available GO gene annotation [see Additional file 1]. This OPI analysis of GO:GNF0006 resulted in a cluster of 37 genes containing 13 of the 18 seed genes (log_10_*P *= -23.0).

### Regulatory Element Discovery using Gene Enrichment Motif Searching

In all, 21 gene clusters representing a diverse set of parasite functions and processes were used as a basis for motif-finding efforts [see Additional file 2]. Intergenic regions in *P. falciparum *are on average 1694 base pairs in length [1]. Therefore, we chose to focus our efforts on finding conserved 5–8 mers motifs located within the 1000 base pairs upstream of gene start codons. Initially, we attempted to apply commonly used motif-finding implementations including MDScan [26] and MEME [24] to our promoter sets, both with and without repeat masking routines, as well as the application of various sequence-trained backgrounds models (see Methods). These efforts resulted in the identification of many motif candidates that were either extremely AT or GC-rich, or found upstream of well over half of the genes in the genome (see MDScan and MEME summary results, [33]). The results of these early efforts led us to the development of GEMS in order to identify more reliably putative *cis*-regulatory elements from the extremely AT-rich and repetitive *P. falciparum *genome.

**Figure 1:**
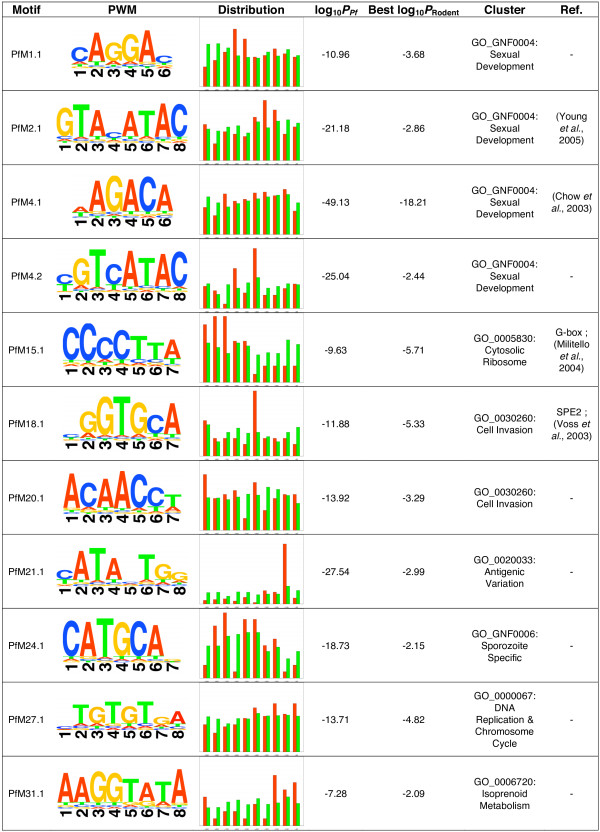
**An overview of some of the most biologically interesting putative regulatory elements identified using GEMS**. Distribution represents the frequency of regulatory elements relative to gene start codons. Red bars represent the location of motifs upstream of genes contained within the cluster (positive set) analyzed whereas green bars represent the location of motifs upstream of genes in the remainder of the genome (negative set). "log_10_*P*_*Pf*_" is the log_10 _of the probability of observed motif enrichment occurring by chance in the positive set versus negative set. "Best log_10_*P*_Rodent_" is the log_10 _probability of observed motif enrichment occurring by change in positive and negative orthologous sets from rodent species (lowest *p*-value from *P. yoelii*, *P. berghei*, or *P. chabaudi *is given).

**Figure 2:**
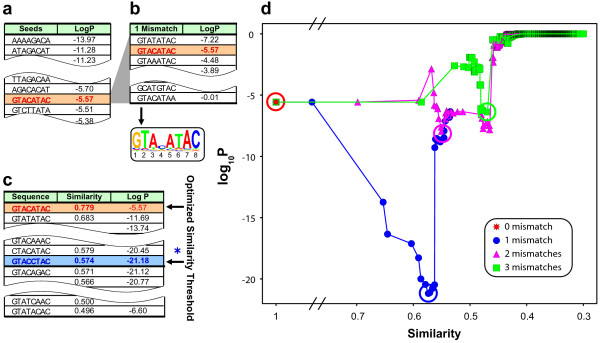
**GEMS identification of PfM2.1 from the Sexual Development cluster (GO:GNF0004)**. a) List of words derived from promoter regions of genes contained within the Sexual Development cluster. The words are ranked by log_10_*P *hypergeometric-derived scores that represent the degree of word enrichment in the promoters of genes contained within the Sexual Development cluster (positive set) versus the remainder of the genome (negative set). In this case, the seed word "GTACATAC" led to PfM2.1 (highlighted in red). b) A re-ordered list of the seed word "GTACATAC" (highlighted in red) and all other words that differ by one mismatch ranked again by log_10_*P *score. A PWM is generated using this list with the contribution of each word to the PWM being weighed by its |log_10_*P*| score. c) A re-ordered list of all words ranked by similarity scores to the generated PWM. Similarity scores for any word are obtained by calculating the geometrical mean of the corresponding PWM elements associated with each word. The similarity threshold that results in the inclusion of words that lead to the lowest *p*-value is identified as optimal (highlighted in blue, blue asterisk). d) Visual depiction of the optimization of parameters through minimization of the *p*-values for different mismatches and similarity thresholds. The local minima corresponding to mismatch 0, 1, 2 and 3 are highlighted by circles (red, blue, magenta, and green respectively). In this case, the optimal log_10_*P *score (-21.2) is found with one mismatch and a similarity score threshold of > 0.57.

To best illustrate how GEMS was applied to each OPI cluster to identify putative regulatory elements, consider the following example outlining the identification of the motif PfM2.1 ("GTACATAC") (Figure 1) from the upstream regions of genes contained within the Sexual Development cluster (GO:GNF0004) (Figure 2). For all the genes contained within a given positive set (the Sexual Development cluster in this example), all unique 5–8 mers (words) occurring in the 1000 base pair defined sequence space were assigned a *p*-value enrichment score using a hypergeometric-based scoring approach (see Methods). This produced a long list of words with associated *p*-values representing the probability of word enrichment in the positive versus negative gene set. These words were then listed in ascending order with the most enriched candidates (lowest *p*-values) serving to seed the construction of PWMs one at a time. In this case, the seed word GTACATAC with a log_10_*P *of -5.57 (highlighted in red) led to the subsequent identification of the putative element PfM2.1 (Figure 2a). Next, all sequences differing from the seed word by one mismatch were identified and re-listed by ascending log_10_*P *values (Figure 2b). A PWM was then generated by individually weighing each word by its log_10_*P *score into the PWM (Figure 2b).

The resulting PWM represents the probability of any given nucleotide occurring at a corresponding location in the putative regulatory element. The similarity of any sequence (8 mer in this case) can be compared to this PWM through the calculation of a similarity score, which is the geometric mean of the corresponding matrix elements associated with the sequence. Next, a similarity threshold was selected to determine how similar any given sequence in a promoter region must be to the PWM to be considered an actual instance of the putative regulatory element. We found from trial-and-error use of various thresholds that this selection is very important for the biological quality of elements eventually obtained. Thus, rather than simply guessing this threshold for each motif, we designed GEMS to utilize an exhaustive parameter optimization routine similar to the probability minimization protocol used in the OPI clustering algorithm [30] to identify optimal similarity thresholds. This was done by first sorting all words by similarity to the PWM (Figure 2c). Then *p*-values were re-calculated as more dissimilar words to the PWM were considered as motif instances (using the same hypergeometric scoring function as was used for the seed scoring) thus identifying the similarity threshold that led to the lowest possible *p*-value (Figure 2c). This entire process was repeated from the original seed word using two and three mismatches up to 40% of the word size to optimize mismatch levels in addition to similarity thresholds (Figure 2d). The similarity and mismatch parameters that resulted in the lowest *p*-value were considered the best representation of a putative *cis*-regulatory element. For PfM2.1, one mismatch with a similarity threshold of 0.57 resulted in the best representation of this putative regulatory element (highlighted in blue, Figure 2c; blue circle, Figure 2d). Lastly, because many seeds led to motif candidates that were subsets of one another, a Tanimoto distance metric that considered positional information was applied to merge non-unique regulatory element candidates (see Methods).

**Figure 3:**
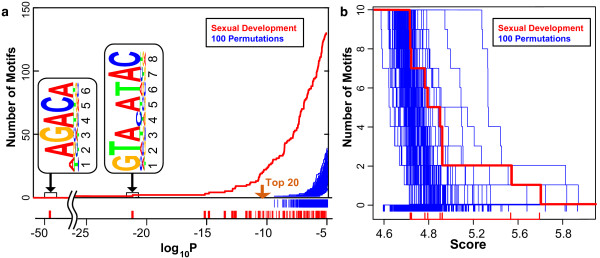
**GEMS and MDScan random permutation analyses**. a) log_10_*P *enrichment score distribution of motif candidates derived from GEMS analysis of the upstream regions of genes contained within Sexual Development cluster (GO:GNF0004) (red). For comparison, the log_10_*P *enrichment score distribution of motif candidates derived from GEMS analysis of 100 randomly selected sets of promoter sequences are also plotted (blue) representing the *p*-value range that is obtainable by chance, i.e. potential false positives. log_10_*P *values for the top 20 motifs from GEMS analysis of the Sexual Development cluster all fall below those obtainable by random simulations. b) MAP score distribution of motif candidates derived from MDScan analysis of the Sexual Development cluster (GO:GNF0004) (red). Again, for comparison, the MAP score distribution of motif candidates derived from MDScan analysis of 100 randomly selected sets of promoter sequences are also plotted (blue) representing the MAP score range that is obtainable by chance. MAP scores for motifs obtained using MDScan analysis of the Sexual Development cluster do not distance themselves from those obtained in random simulations suggesting the potential for many false positives in MDScan motif discovery.

For each cluster, this GEMS analysis resulted in a large number of putative regulatory elements possessing widely varying *p*-values. In order to determine the *p*-value range most likely to represent true positives, we performed GEMS analyses on 100 independently selected random positive sets of promoter sequences to estimate the likelihood of obtaining any given *p*-value by chance. These exercises demonstrated that *p*-values for motifs identified from these random promoter sets were consistently higher than 10^-8^, whereas in comparison *p*-values for the top motifs identified using a promoter positive set derived from the Sexual Development OPI cluster (GO:GNF0004) were well below 10^-10 ^(Figure 3a). These results suggested biological relevance for the top scoring motifs identified by GEMS using OPI cluster-derived promoter sets, as similar *p*-value scores could not be obtained in the absence of biological-based co-expression data (permutation simulations). In contrast, when MDScan was applied to the same Sexual Development OPI cluster positive promoter set and randomly selected positive promoter sets, the delimitation between scores for motifs identified from the true positive set and 100 random positive sets was much less pronounced (Figure 3b). This indicates the best scoring motifs identified using MDScan may not be necessarily biologically relevant as similarly scored motifs can be obtained from randomly selected sets of sequences. Application of MEME to the same cluster resulted in motifs with extremely high E-values or very insignificant group-specificity scores.

Judging from the results of the GEMS analyses of random positive promoter sets, the top 20 motifs possessing the lowest *p*-values from each of the 21 clusters were retained for completeness (420 motifs) [33]. To further pare down the list of candidate motifs to a more manageable number for subsequent biological analysis, we identified those motifs that were most conserved in the upstream regions of orthologous rodent *Plasmodium *parasites species genes. To do this, the orthologs of *P. falciparum *genes were identified in *Plasmodium yoelii*, *Plasmodium berghei*, and *Plasmodium chabaudi *and the degree of motif enrichment in these orthologous gene upstream regions were calculated using the hypergeometric function just as was done in *P. falciparum*. In general, these *p*-values were less significant due to the fact that only ~60% of the genes in *P. falciparum *have clear rodent orthologs. For motifs to be further considered as putative *cis*-regulatory elements, we required them to have an enrichment score of log_10_*P *≤ -2 in at least one of the three rodent malaria species. This filtering step resulted in 50 evolutionarily conserved putative *cis*-regulatory elements [see Additional file 3]. Since the GEMS analysis is run in a parallel fashion for all OPI clusters and because some of these OPI clusters represent processes with similar expression profiles, some of the 50 motifs contained within this list represented the same regulatory element rediscovered from multiple OPI clusters. As a result, the 50 motifs were clustered one final time using a CompareACE-equivalent algorithm with a similarity cutoff of 0.8 resulting in a final list of 34 unique and high-confidence putative *cis*-regulatory motifs candidates [see Additional file 3]. Motifs that were clustered together in this process were labeled PfM#.1, PfM#.2, PfM#.3, etc., with the lowest *p*-value candidate being considered the final best putative *cis*-regulatory element.

**Figure 4:**
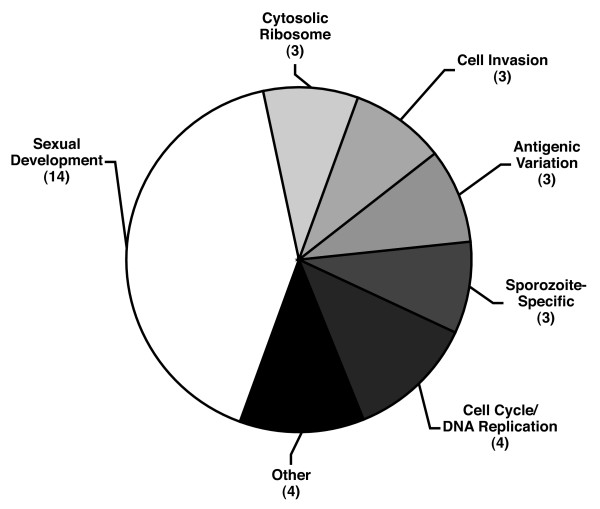
Promoter-derived putative regulatory elements as discovered by OPI cluster.

The final list of 34 best representative motif candidates originated from a diverse array of clusters (Figure 4). In addition to the many novel motifs, most of the previously described motifs from the literature were also rediscovered demonstrating the robustness of GEMS analysis on OPI-generated gene clusters for elucidating transcriptional control mechanisms within the context of the AT-rich *P. falciparum *genome (Figure 1). As an additional metric for the biological significance of each motif, the distribution of motifs found upstream of genes within the seed clusters was plotted side-by-side with the positions of the motifs found upstream of all other genes in the genome as biologically active motifs are likely to exhibit some positional enrichment with respect to the start codon [25]. For example, PfM1.1 derived from the Sexual Development cluster (GO:GNF0004) exhibited a positional enrichment 600–799 base pairs upstream of the start codon of genes contained within the cluster suggesting for this element to be functional, its positional context may be important (Figure 1).

### Gene Enrichment Motif Searching Analysis of Gene Introns

As it is known that *cis*-regulatory elements also sometimes reside within gene introns, we also applied GEMS to search for putative regulatory elements within these areas of the *P. falciparum *genome. Overall, we used a similar approach as was taken for the GEMS analysis of upstream regions, but with a few minor modifications. In all, 54% of *P. falciparum *genes possess introns, with the average length of these regions being 178.7 base pairs [1]. Thus, to generate an intron search space, all exons were masked out from the differentially expressed genes sequences leaving intron sequences from a total of 1620 genes. To accommodate for the variable lengths of introns in each gene, the hypergeometric calculation of motif *p*-values was slightly altered to use base pair counts instead of gene counts (see Methods). GEMS analysis of intron regions resulted in the identification of 130 motifs in total [34]. Then, for intron-derived motifs to be further considered as putative *cis*-regulatory elements, motifs were required to have an enrichment score of log_10_*P *≤ -3 in *P. falciparum*, and log_10_*P *≤ -2 in at least one of the three rodent malaria species. This filtering step resulted in 32 evolutionarily conserved putative *cis*-regulatory elements. Again, due to the parallel fashion in which GEMS was run, some of these 32 motifs represented the same regulatory element rediscovered from multiple OPI clusters and so a CompareACE-equivalent algorithm with a similarity cutoff of 0.8 was applied to obtain a final list of 26 intron-derived *cis*-regulatory motifs candidates. These candidates were labeled PfMIntron#.1, PfMIntron#.2, etc with the lowest *p*-value candidate being considered the final best putative *cis*-regulatory element [see Additional file 4].

### Sexual Development Regulatory Elements

Of all the gene clusters analyzed, the sexual development cluster (GO_GNF0004) yielded the most upstream region motif candidates (14 of 34) (Figure 4). This cluster contains genes whose expression is upregulated as the parasite prepares to transition from the human host to the mosquito vector as it was derived from expression analysis of a mixed population of developing male and female gametocytes. For two of these motifs, existing biological evidence from the literature suggested they played roles as *cis*-regulatory elements of transcriptional control (Figure 1). For example, the best scoring motif, PfM4.1 (WAGACA, log_10_*P*_*Pf *_= -49.13) (Figure 1), is contained within the longer sequence CAGACAGC present in the promoter of *pgs28*, a gene encoding a major surface antigen in the avian malaria parasite, *P. gallinaceum*. Using linkage scanner mutagenesis and EMSAs, Chow and co-workers demonstrated that disruption of this element reduced luciferase reporter expression by 65% in *P. gallinaceum *ookinetes and that unknown *trans*-acting factors present in *P. gallinaceum *ookinete nuclear extracts bound this element in a sequence-specific manner [35]. Further investigation of PfM4.1 on our part also revealed that this motif is very well conserved in the rodent malaria parasite species *P. yoelii *(log_10_*P*_*Py *_= -18.21), *P. berghei *(log_10_*P*_*Pb *_= -9.89), and *P. chabaudi *(log_10_*P*_*Pc *_= -9.93) and that it is enriched in the promoter regions of GO annotated microtubule-based movement genes in *P. falciparum *(log_10_*P *= -3.91). The core sequence AGACA was also present in the promoters of 14 of the 21 *P. falciparum *male-specific *P. berghei *gametocyte genes, but only 2 of the 25 *P. falciparum *female-specific *P. berghei *gametocyte genes identified in a recent proteomic analysis performed on *P. berghei *male and female gametocytes [36]. Examples of some of these male-specific genes included dynein-associated protein (PF14_0202), dynein light chain (PF11_0148), dynein heavy chains (PF10_0224, PF11_0240) and kinesin (PFA0535c) suggesting that PfM4.1 may play a potential role in regulating the production of the microtubule-rich flagellum specific to male gametocytes. Furthermore, because we also observed that although PfM4.1 occurs at a moderate frequency in the genome (1836 times in the genome), with many genes containing the motif possesses two or more copies (1.9 copies on average), cooperative binding of *trans*-factors within the proper genomic context may be important for the proper function of this motif.

### Cell Invasion Regulatory Elements

**Figure 5:**
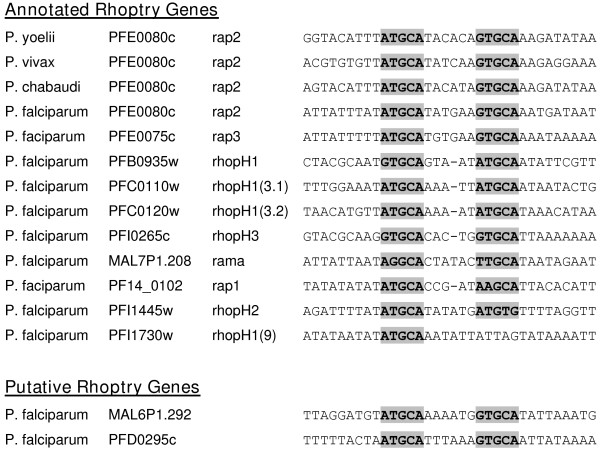
**Alignment of sequences from known and putative rhoptry gene promoter regions containing PfM18.1 and PfM18.1-like motifs**. Most genes have two copies separated by six bases (highlighted in grey and bold).

Malaria parasites possess unique sub-cellular organelles (i.e. rhoptries and micronemes) that perform parasite-specific functions during red blood cell invasion. Studies in *S. cerevisiae *have demonstrated that genes whose proteins are co-localized to macromolecular complexes or sub-cellular organelles such as the ribosome often exhibit tight transcriptional co-expression regulated by similar *cis*-regulatory elements [37]. Thus, we hypothesized that co-expressed genes whose products are recruited to these parasite-specific sub-cellular complexes might also be regulated by common *cis*-regulatory elements. Analysis of the cell invasion cluster revealed three candidate motifs (Figure 4) of which PfM20.1 (ACAACCT, log_10_*P*_*Pf *_= -13.92) and PfM18.1 (NGGTGCA, log_10_*P*_*Pf *_= -11.88) (Figure 1) were of particular biological interest. PfM20.1 was found upstream of five of the eight characterized microneme genes encoding proteins localized to the peripheral surface of the merozoite including the apical membrane antigen AMA-1 [38]. PfM18.1 was found to be associated with genes encoding proteins localized to the rhoptries including the rhoptry-associated proteins (RAPs) RAP1 and RAP2. PfM18.1 was also well conserved in upstream regions of homologous genes in the rodent malaria species *P. yoelii *and *P. berghei *(log_10_*P*_*Py *_= -5.33; log_10_*P*_*Pb *_= -3.70) as well as upstream of the *P. vivax rap2 *gene (Figure 5). Closer inspection of all the annotated rhoptry genes in *P. falciparum *revealed that highly similar sequences to PfM18.1 were found within 1200 bases upstream of the start codon of nine of the ten rhoptry genes described by Cowman *et al*. [38] and that in all but one instance, two copies of this motif were separated by six or seven nucleotides, GTGCA(N_5–6_)GTGCA (Figure 5). Other genes expressed during schizogony bearing this larger dyad motif were the uncharacterized genes MAL6P1.292 and PFD0295c, both of which encode homologous proteins to components of the *Toxoplasma gondii *rhoptries as identified by proteomic analysis [39] (Figure 5). Furthermore, the motif is generally not found upstream of other genes expressed during the invasive stages of the erythrocytic cell cycle that are localized to different cellular locations (an exception being the S-antigen, which is thought to be localized to the peripheral surface of the invasive merozoite) indicating this motif is likely involved in rhoptry-specific gene expression.

A version of PfM18.1 was previously identified as the SPE2 motif in a study on *var *gene expression [8]. *Var *genes, of which there are approximately 60 in the *P. falciparum *genome, encode extremely antigenically variable erythrocyte membrane-localized proteins that are subject to complex mechanisms of gene silencing [40]. Using EMSAs and promoter deletion mapping, Voss and co-workers demonstrated that the SPE2 motif is bound by a protein in the late stages of the intraerythrocytic cycle (>34 hours post infection) and that elimination of the SPE2 motif repeat array resulted in an approximately 2-fold increase in promoter activity [8]. The authors interpreted these data to suggest that the SPE2 motif serves as part of a silencing mechanism as *var *genes are exclusively transcribed during the late ring stages of the intraerythrocytic cycle [13,14]. However, the schizont-specific EMSA shift of the SPE2 motif also supports our GEMS results that suggest PfM18.1 is the binding site for a rhoptry-specific transcriptional activator potentially responsible for activation of cell invasion genes during the late stages of the intraerythrocytic cell cycle. The notion of a binding protein serving a dual role in *var *gene silencing and rhoptry-gene activation has precedent in the literature as in the case of the yeast repressor-activator protein RAP1 which both activates and silences transcription of mating-type genes in *S. cerevisiae *[41,42]. Furthermore, the SPE2 motif identified by Voss and co-workers was found in tandem copies approximately 2500 bases upstream of telomeric *var *genes unlike the 400–499 base pair location of most of the PfM18.1 instances we found using GEMS suggesting location of the motif relative to gene transcription start sites may be of importance for biological function (Figure 1).

**Figure 6:**
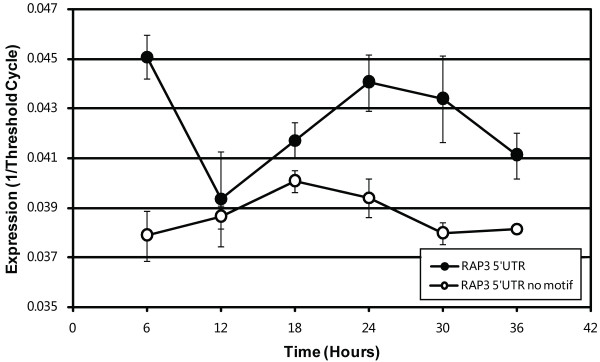
**Results of transient transfection using 1527 base pair promoter region of *rap3 *cloned upstream luciferase reporter as monitored by qRT-PCR**. The native promoter (black) results in a life cycle stage-specific expression pattern while specific deletion from the construct of the two copies of PfM18.1 and interspersed sequence at -588 base pairs (white) eliminates this effect.

We further investigated the role PfM18.1 may play in RAP gene expression using transient transfection luciferase reporter gene assays and EMSAs. Specifically, we chose to explore the role PfM18.1 played in RAP3 (PFE0075c) expression because this gene had a simple structure with no introns making the identification of the true start codon relatively straightforward. Transient transfection of synchronized parasites with a luciferase construct driven by the native ~1500 base pair promoter region upstream of the RAP3 start codon resulted in expression pattern that was consistent with previously published microarray data (Figure 6) [13]. However, precise deletion of the two copies of PfM18.1 and interspersed sequence the PfM18.1 at 588 base pairs upstream of the RAP3 start codon abolished of distinct stage-specific expression pattern demonstrating PfM18.1 is essential for proper control of transcript levels (Figure 6).

**Figure 7:**
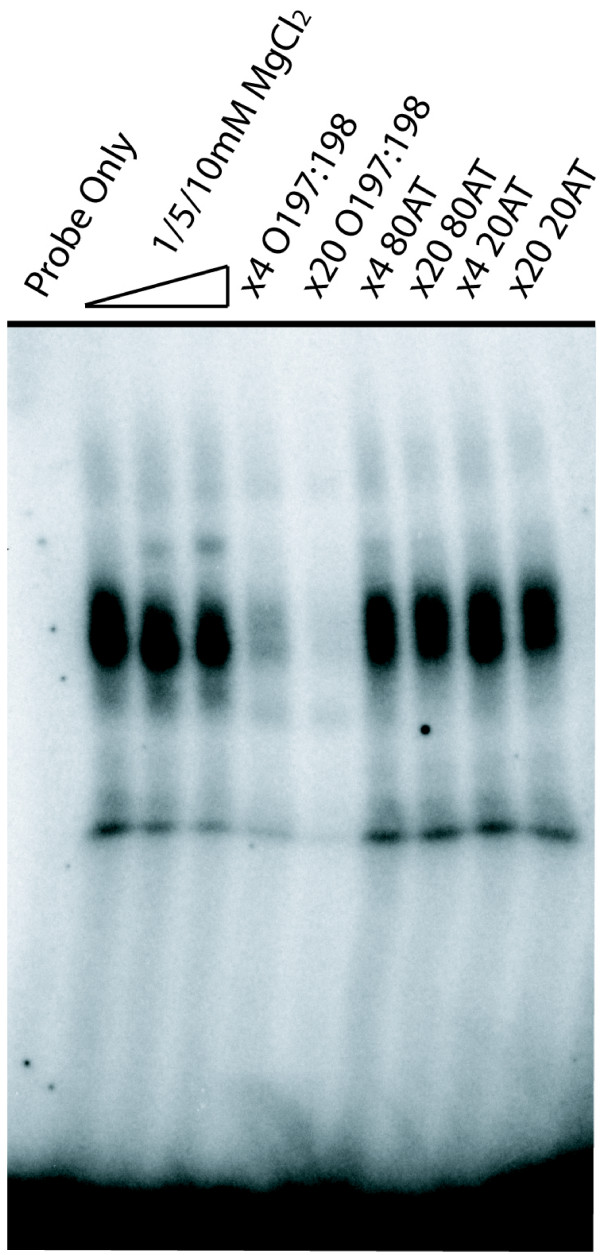
**Characterization of PfM18.1 binding proteins by EMSA**. Incubation of ^32^P radiolabeled-probe containing PfM18.1 generated a multi-complex shift (O197:198, Lane 2). ×4 and ×20 molar excess cold competitor O197:198 diminished the shift in a concentration dependent manner (Lanes 5 & 6) while random 80% AT (Lanes 7 & 8) and random 20% AT (lanes 9 & 10) cold competitor probes did not compete indicating sequence specificity for the binding event. ×5 and ×10 increase in MgCl_2 _concentration resulted in intensification of the second-most upper band (Lanes 3 & 4).

Next, we performed EMSAs to investigate if PfM18.1 was bound by *trans*-acting factors. Specifically, a 34-mer oligonucleotide probe containing PfM18.1 and surrounding sequence from the RAP3 promoter was radiolabeled using Klenow fragment and incubated with nuclear extracts isolated from mixed asexual stage parasites. This resulted in formation of several shift complexes that could be competed away with cold probe of identical sequence in a concentration dependent manner (4 and 20-fold excess), but not by random 80% AT and 20% AT probes suggesting sequence specificity for this binding event (Figure 7). Furthermore, the multiple shifts diminished equally with addition of competitor indicating that the various observed complexes are most likely the result of a disassociation of protein complex or proteolysis. Overall, these data support the hypothesis that PfM18.1/SPE2 is an activator of rhoptry gene expression in addition to its role as a possible silencer of *var *gene expression.

### Protein Biosynthesis Regulatory Elements

Analysis of the gene cluster "Cytosolic Ribosome (sensu Eukarya)" resulted in the identification of three motifs (Figure 4) of which the most statistically significant was PfM15.1 (CCCCTTA, log_10_*P*_*Pf *_= -9.63) (Figure 1). PfM15.1 is positionally enriched occurring in highest frequency between 700 to 999 bases upstream of gene start codon (Figure 1). This is in contrast with motifs identified from other processes such as sexual development or cell invasion where motif distributions are generally found predominately in the range of 300 to 700 bases. There was also evolutionary support for this motif as it was well conserved in the rodent malaria species *P. chabaudi *and *P. yoelii *(log_10_*P*_*Pc *_= -5.71, log_10_*P*_*Py *_= -4.15). This motif was also the approximate reverse complement of the "G-box" motif (ATGGGGC) previously discovered by Militello and co-workers using an AlignACE analysis of the promoter regions of annotated *P. falciparum *heat shock genes [9].

**Figure 8:**
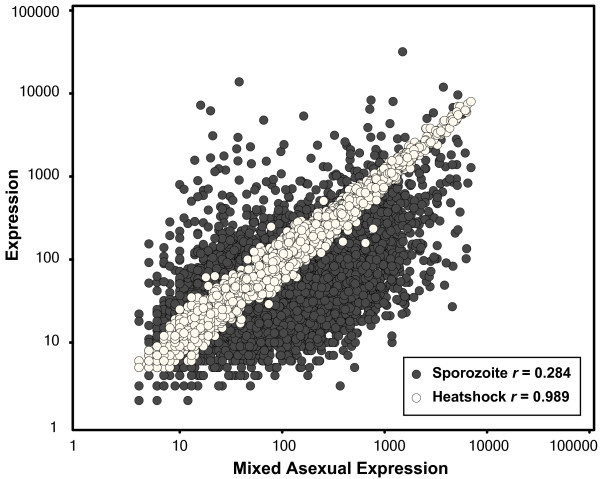
**Gene-by-gene comparison of expression levels for mixed asexual versus sporozoite stages and mixed asexual parasites versus heat shock treated mixed asexual parasites**. While expression levels for many genes vary widely between mixed asexual and sporozoite stages (dark gray points, Pearson's *r *= 0.284), little difference is observed in expression levels in mixed asexual parasites before and after heat shock treatment (white points, Pearson's *r *= 0.989) demonstrating a lack of robust transcriptional response to environmental perturbations at the level of transcription.

A long-standing question in malaria research is whether environmental or drug stimuli trigger transcriptional responses in genes active in the affected biological pathways. In *S. cerevisiae*, specific components of signaling pathways have been identified using a DNA microarray-profiling approach to analyze the yeast transcriptional responses to various drug pressures [43-45]. If *P. falciparum *behaves in a similar fashion to yeast, insights could be gained into the biological targets of certain anti-malarial drugs for little is known regarding specific modes of action. Since PfM15.1 was originally identified as the G-box motif using heat shock annotated genes, it provided us with an opportunity to test the biological specificity of transcriptional responses in *P. falciparum *to heat shock. If the application of heat shock resulted in a specific transcriptional response mediated by a *cis*-regulatory element, we hypothesized that we would be able to re-identify PfM15.1 from a list of differentially expressed genes obtained from experimental heat shock transcriptional response data. To test this hypothesis, we exposed a mixed asexual culture *P. falciparum *to a 42°C heat shock for one hour and identified differentially expressed genes relative to a negative control by hybridization to our whole genome high-density microarray. The greatest fold change for any one gene between heat shock and negative control was 4.03 demonstrating that transcriptional responses to environmental stimuli are not as robust in *P. falciparum *as has been reported in other organisms such as yeast and humans (Figure 8). However, by defining the top 75 differentially expressed genes identified by a non-parametric Mack-Skillings as a positive set [see Additional file 5] and searching the 2000 bases upstream of these genes, GEMS analysis was able to rediscover a motif similar to PfM15.1/G-box motif (ATGGGGCC, log_10_*P*_*Pf *_= -5.06) [46]. Although this motif was the 12^th ^best scoring motif in this analysis, the fact that GEMS was able to rediscover the G-box equivalent motif even from this modest heat shock transcriptional response was encouraging. Since versions of PfM15.1 were also discovered from clusters of genes that show similar expression patterns to ribosomal genes, namely mitotic cell cycle (PfM15.2, AAAGGGA, log_10_*P*_*Pf *_= -8.72) and tRNA metabolism (PfM15.3, TAGGGGAA, log_10_*P*_*Pf *_= -7.41) [see Additional file 3], as well as from intron regions of genes involved in tRNA metabolism (PfMIntron13.2, CCTCCCCC, log_10_*P*_*Pf *_= -3.29) and the ribosome (PfMIntron16.2, ACGGGGG, log_10_*P*_*Pf *_= -3.91) [see Additional file 4], it is possible that in general the G-box motif is primarily associated with highly expressed trophozoite-specific metabolic genes and that identification of the G-box through heat shock gene promoter analysis by Militello *et al*. was merely fortuitous as heat shock genes are highly-expressed during these same stages.

### Antigenic Variation Regulatory Elements

In *P. falciparum *there are several gene families (*var*, *rifin*, *stevor*) that encode proteins expressed on the surface of infected red blood cells that are important for parasite cytoadherance and virulence. Although the coding regions of members of these gene families are extremely variable enabling the parasite to switch expression of isoforms of these surface proteins to successfully evade host immune system detection, the promoter regions of these are remarkably conserved [47]. It is therefore necessary to take great care in interpreting the results of any GEMS analysis of promoters of these genes as the exceptionally high-scoring motif candidates can result simply because the promoter regions of these antigenic variation genes are duplicated across members of the same gene family. For instance, the top-scoring motif PfM21.1 (CATANTGG, log_10_*P*_*Pf *_= -27.54) (Figure 1) obtained from analysis of the Antigenic Variation cluster (GO:0020033) was identified because large swaths of the promoter regions of these antigenic genes were conserved. This was illustrated by its strong positional enrichment 100–199 base pairs upstream of gene start codons. In another case however, the motif PfM4.2 (CGTCATAC, log_10_*P*_*Pf *_= -25.04) (Figure 1) was found upstream of members of the *rifin *and *var *gene families in regions where sequences upstream or downstream were not conserved as represented by a still present, yet less pronounced, positional enrichment 400–499 base pairs upstream of gene start codons. Therefore, while positional enrichment of motif location relative to start codons can be used as functional support when the motif is derived from genes of multiple functions, a high-degree of positional enrichment can also be used to identify a motif as a potential artifact if the genes come from a highly-duplicated gene family as is the case for antigenic variation genes.

### Sporozoite-specific Regulatory Elements

The sporozoite stage of the parasite life cycle is the subject of intense investigation because transcripts or proteins present during this stage are likely responsible for the effectiveness of the irradiated sporozoite malaria vaccine [48]. The most significant motif GEMS identified associated with sporozoite-stage gene expression was PfM24.1 (CATGCAN, log_10_*P*_*Pf *_= -18.73) (Figure 1). PfM24.1 was found in the promoters of 31 of the 37 genes in the Sporozoite-Specific cluster (GO:00006) including the well-known genes sporozoite surface protein 2 (TRAP), CSP, and MAEBL (Table 1). Both TRAP and CSP are major vaccine targets and identifying other genes that are co-regulated could point to additional vaccine targets. Furthermore, CSP appears to be involved in host immune modulation so that the transcription factors that bind to the element and control the expression of this gene could represent promising drug targets. Interestingly, two other genes whose promoters contained PfM24.1 included the genes succinate dehydrogenase and citrate synthase that are nearly adjacent in the mitochondrial tricarboxylic acid (TCA) cycle. Both these genes, as well as other components of the TCA cycle are for the most part highly expressed in sporozoites and gametocytes [14,32], therefore suggesting an active TCA cycle during the insect stages of the parasite life cycle [see Additional file 6].

**Table 1:** Alignment of promoter regions from sporozoite-expressed genes containing PfM24.1.

**Locus**	**Description**	**Start**	**Sequence**	**End**
MAL13P1.125	-	-312	tatatttttttttataagga**CATGCAC**ttaatttttaaaagattttc	-358
MAL13P1.212	-	-767	ttttaaaatttttcttaaag**CATGCAC**aattaagaagacgaaaaaac	-813
MAL8P1.6	-	-55	ttttttttttcttagttata**CATGCAA**aataaataaataagtttata	-9
PF08_0088	S23	-486	caaattttttttttctcctg**CATGCAG**catttatatattaacttcaa	-532
PF08_0088	S23	-529	aagttaatatataaatgctg**CATGCAG**gagaaaaaaaaaatttgatt	-483
PF10_0218	citrate synthase	-936	tagctcaaccaaaacataag**CATGCAA**tatatttttatacttttata	-890
PF10_0231	-	-427	tttaaatcttcataccaaca**CATGCAC**aaaagaaataaaaaattaca	-381
PF11_0328	-	-756	tatattataaatactctctg**CATGCAA**tatatatgtatattactcca	-710
PF11_0480	S22	-131	ttgtcctatccaaaaattga**CATTCAG**agttatagaaaaaaaatata	-85
PF11_0486	MAEBL	-828	attttcttcatataagaaca**CATGCAG**atttttattattgtctattt	-874
PF13_0201	SSP	-851	gaatcagatttattcaaacg**CATGCTG**ttttaaaataaaaaaaaaaa	-897
PF14_0074	-	-837	atataaagctacaatacacc**CATGCAT**caacaatatatcctattttg	-791
PF14_0074	-	-449	tctgttttttttgtcattta**CATGCAT**tttatggttgtaaaatgaac	-495
PF14_0427	-	-414	aaaaaaaaaaaatatacata**CATGCAT**acataagtttctatttttca	-460
PF14_0427	-	-325	aaaattttgtatgtattata**CATGCAT**attattataatatggtttta	-279
PF14_0729	-	-521	tttgtccatttataaattag**CATGCAA**tctatgtctgatattaaaca	-475
PFA0200w	TSRP	-265	ataattacatatttggtcta**CATGCAT**atacaagacaatatattgta	-219
PFA0205w	S24	-900	aatgaacatatattagctta**CATGCAA**aaagaaaaaaaatatatatt	-854
PFA0380w	Kinase	-986	ttttttttttttttttgaaa**CATGCAT**tgttttattcttaaaaaata	-940
PFB0325c	SERA	-691	atttatgagctgaattgtta**CATGCAT**taaaaatggcaatgggaaat	-737
PFC0210c	CSP	-708	tgggattattgtaaatataa**CATGCAC**attttgtataagttccttaa	-662
PFC0210c	CSP	-606	cagaaattattcttatctta**CATGCAC**atataaaaaaatggattggt	-560
PFD0215c	Pbs36	-399	gtgagttctacatgccactg**CATGCTG**atataaaataaattaaaaaa	-353
PFD0235c	S10	-471	tatctaggcacgtttcatca**CATGCAT**aaaaaaataaaaaataaaaa	-517
PFD0425w	S17	-897	gattgttatatttatcgtta**CATGCAT**gtttcctaattttttttttt	-851
PFD0425w	S17	-850	aaaaaaaaaaaattaggaaa**CATGCAT**gtaacgataaatataacaat	-896
PFD0430c	Ppl1	-578	ttttttttaaataaatcatg**CATGCAC**atataattatatttttgtct	-532
PFE0360c	s14	-915	aataacatcttgtattgtca**CATGCAA**aatgaaattatcacatttat	-869
PFE0565w	-	-765	tatatcaaaaacgagacatg**CATGCAA**ttcattttgattcaattgtt	-719
PFE0950c	-	-396	tttccattttttttcctgaa**CGTGCAG**gaatttaaatattattaatt	-350
PFL0065w	-	-283	aaaaaagaaaccataatatg**CATGCAC**atattaataaatatatatat	-237
PFL0370w	-	-505	aactctctttttttatataa**CATGCAT**gttgtagagctgtatataaa	-459
PFL0370w	-	-458	ttttatatacagctctacaa**CATGCAT**gttatataaaaaaagagagt	-504
PFL0630w	-	-376	atatattattaaaatgtgat**CATGCAA**aaaaaatgaaaaaacattat	-330
PFL0800c	S4 succinate dehydrogenase	-804	tttttttcgctttatttatg**CATGCAA**ataagttgatattcctgaac	-758
PFL1770c	-	-748	ctgatatataataatggggt**CATGCAG**gtattgaaatacacttaaaa	-794
chr1.rRNA-1-28s	-	-868	aatagtatcggtgtaattta**CATTCAG**cattctgatgatttacagta	-914

Motifs are highlighted in bold. Start and stop locations are relative to start codons.

### Cell Metabolism and DNA Replication Regulatory Elements

Motifs associated with cell metabolism and DNA replication genes overall were less striking. For example, PfM27.1 (NTGTGTGA, log_10_*P*_*Pf *_= -13.71) (Figure 1), identified from the DNA Replication and Chromosome Cycle cluster (GO:000067), was found relatively ubiquitously potentially because it is involved in the regulation of a large cohort of genes expressed during middle to latter parts of the intraerythrocytic life cycle. Another motif of slightly greater interest was PfM31.1 (AAGGTATA, log_10_*P*_*Pf *_= -7.28) (Figure 1), which was identified from the Isoprenoid Metabolism cluster (GO:0006720). This motif was found upstream of four of the seven annotated isoprenoid metabolism pathway genes [49]. Isoprenoid metabolism occurs in the apicoplast, a plastid organelle unique to Apicomplexans that is involved in the synthesis of lipids and heme [50]. Thus, transcription factors that regulate this entire pathway through PfM31.1 may serve as promising drug targets for the inhibition of this entire parasite-specific metabolic pathway.

### Key aspects of Gene Enrichment Motif Searching

The two aspects of GEMS that appeared critical to its success in *P. falciparum *were: 1) the use hypergeometric scoring function based on empirical sequences derived from highly co-regulated OPI gene clusters; 2) mismatch and similarity threshold optimization routine that objectively identified optimal PWMs. On the first point, when dealing with repetitive and base-biased genomes such as *P. falciparum*, hypergeometric-based scoring approaches seem to have an advantage over background-modeling approaches because they compare the frequency of full motifs occurring in empirically derived positive and negative sequence sets rather than merely relying on an estimation of background nucleotide frequencies. Thus, with hypergeometric-based approaches, repetitive sequences present both in positive and negative sets cancel one another out where many low-order background-modeling approaches would identify the same repetitive sequences as potential motifs because they vary significantly from the background estimation. Indeed, it has been previously observed that hypergeometric-based scoring functions in general tend to produce better *p*-value scores in motif ranking criterion when compared to light-weighted MAP functions [51]. It should also be noted that we are not the first to use hypergeometric-based scoring approaches for motif discovery in *P. falciparum*. Militello and co-workers previously applied the AlignACE algorithm, which uses a hypergeometric-based scoring function for efficient post-processing of motif candidates scores [25,51,52], to identify the G-box element in the promoter regions of hand-selected set of *P. falciparum *heat shock genes [9]. Similarly, van Noort and co-workers applied a more sophisticated AlignACE-based approach to identify 28 highly-abundant putative regulatory elements in the *P. falciparum *genome [53].

The second key to the success of GEMS appeared to be the implementation of the automated mismatch and similarity threshold optimization routine that objectively identified optimal PWMs. A PWM without a similarity threshold is meaningless because it is simply a probabilistic description of base frequency for a motif based on collection of possible *cis*-regulatory elements. As a result, many motif-finding algorithms that employ PWMs to represent regulatory elements require manual selection of similarity thresholds, which can be an arbitrary and tedious process. However, selection of these thresholds is critical to obtaining quality PWMs that best represent regulatory elements whose enrichment in positive gene sets can best explain the co-regulation of the gene cluster. By using an OPI-like parameter optimization routine, much of the arbitrariness of similarity and mismatch threshold determination is bypassed and PWMs are optimized based on a biologically relevant *p*-value minimization rather than by often times vague user assumptions. This is especially important for organisms about which little is known regarding regulatory elements since in these cases it is difficult to minimize false positives and negatives through element rediscovery trial and error due to lack of information. The optimization routine is also automated, which is particularly important when GEMS is applied to many gene clusters resulting in 100s to 1000s of motifs for which manual determination of similarity and mismatch thresholds would be impractical.

### False positives and false negatives

As with any *in silico *regulatory element discovery process, the possibilities of false positives and false negatives was always considered during our analysis. Although false positives are best addressed by analyses of randomly selected promoter sequences (as depicted in Figure 3) and the consideration supporting evidence such as evolutionary conservation or motif positional enrichment relative to start codons to identify those highest confidence candidates for validation using more traditional experimental techniques, there are several improvements that could be made in the future to potentially lower the false negative rates. A recent analysis of *P. falciparum *cDNA libraries revealed that an estimated ~24% of *P. falciparum *open reading frames may have errors in their annotated structure [54]. As a result, the delimitation of the promoter sequences we chose in this study in some instances may have been less than ideal. In the future, a better approach would be to use transcription start sites instead of start codons to define upstream promoter sequences for motif discovery. For this study, however, comprehensive information was not readily available for *P. falciparum *as attempts to map transcriptional start sites have yielded ambiguous results [4]. The inclusion of transcriptional start sites would serve to improve accuracy of our GEMS analysis since it would allow the better distinction between conserved elements that are potentially bound by the core transcriptional complex versus those that act as enhancer/repressor binding sites. It is also possible because we focused our search space primarily on the 1000 bases upstream of start codons that elements presented herein may be an overrepresentation of core transcriptional complex binding sequences. Lastly, another potential disadvantage of the GEMS is that it is not well suited for discovery of long motifs that may be separated by spacer elements. We attempted to search for larger motifs as part of the GEMS analysis by searching for associations between individual 5–8 mers due to their physical linkage on the chromosome as was done in the rediscovery of the GAL4 binding site (CGG(N_11_)CCG) in yeast [55]. However, applying this type of method in *P. falciparum *did not reveal many larger sites that were more impressive than the half sites alone, with the notable exception being the cell invasion-related motif PfM18.1.

Beyond the consideration of the promoter input sequences and motif search spaces, improvements in the clusters provided to GEMS for analysis will likely also improve our false negative discovery rate in the future. We primarily relied on GO annotations as the basis for the OPI clustering of genes showing differential expression across the parasite life cycle. As our understanding of *P. falciparum *gene function improves and as additional expression profiles from the liver, early sporozoite and mosquito midgut stages of the parasite life cycle are obtained, the quality of OPI clusters will improve thus providing better starting points for GEMS analysis. Having expression data from related parasite species will also likely improve confidence in genes that are expressed at low levels, resulting in larger sets of co-expressed genes and higher-confidence motif detection. While cDNA and some limited microarray data are available for some of these stages and species at present, we have not used this information to date because these data were collected on a variety of different platforms using different normalization methods and to different degrees of completeness thus making their incorporation into our algorithms difficult. Lastly, although in this study we only considered life cycle expression data in the main analysis, the inclusion of microarray data of transcriptional responses to various drugs or conditions could also be used for the basis of OPI clustering and GEMS analysis to elucidate components of drug response pathways. In the future we aim to incorporate data acquired from these *P. falciparum *life cycle stages and drug treatments as well as corresponding rodent malaria expression data using a high-density microarray platform.

## Conclusion

*In silico *approaches of regulatory element discovery promise to serve as a rapid and efficient means for generating high quality hypotheses for further biological investigation in organisms where little is known regarding transcriptional control mechanisms. We have demonstrated through the development of GEMS and its application to OPI-generated microarray gene clusters that *in silico *regulatory element discovery is indeed feasible for even the most challenging repeat-sequence-rich base-biased genomes, such as that of *P. falciparum*. By utilizing a hypergeometric-based scoring function and a PWM optimization routine, GEMS predicted with high-confidence 34 putative regulatory elements from the upstream regions of genes contained within a diverse array of functional gene clusters, thus supporting the hypothesis that *cis*-regulatory elements play an important role in the transcriptional control of many *P. falciparum *biological processes. We also provided biological support for the importance of one cell invasion-related element (PfM18.1) by EMSA and reporter gene assay thus demonstrating that these putative elements hold much promise for further biological characterization. Overall, the many putative regulatory elements described herein will serve as much-needed starting points for future decoding of the currently poorly understood basis of gene regulation in malaria parasites. Knowing precisely how transcription is controlled in these systems will improve our ability to genetically engineer parasites, test hypotheses about gene function, and eventually develop novel therapeutics against malaria.

## Methods

### Transcriptome Microarray Datasets

Three microarray datasets generated using the custom-designed *P. falciparum *full-genome high-density oligonucleotide array (Affymetrix, USA) containing 25-mer probes to 5159 *P. falciparum *genes [14] were analyzed in the course of this study. The first dataset contained 16 time points covering the various parts of the parasite life cycle including seven sorbitol synchronized asexual cell cycle stages, seven temperature synchronized asexual stages, a single sporozoite stage sample, and a single late-stage gametocyte sample [14]. The second dataset was comprised of 23 time points from three more detailed day-by-day time-courses of gametocyte development, including high-purity early stage gametocytes [32]. The third dataset was generated for the purposes of this study and contains information on the heat shock transcriptional response of *P. falciparum *parasites. For this, *P. falciparum *clone 3D7 was cultured in a 36°C incubator using human O+ erythrocytes as previously described [56]. At a parasitemia of ~12% as assessed by Giemsa staining of blood smears, the culture medium was removed by aspiration and 42°C medium or 36°C medium was added to cultures to initiate heat shock and negative control treatment respectively. The cultures were then incubated at 42°C (heat shock) and 36°C (control) for 1 hr. At the end of 1 hr, parasite total RNA was isolated immediately by addition of Trizol reagent addition to cultures as previously described [57]. Labeling of the RNA and hybridization to the above described high-density microarray was conducted as previously described [14]. The raw data files from this match-only microarray were analyzed using the Match-Only Integral Distribution algorithm (MOID) [58]. Background correction and normalization were carried out using the same processing protocol as previously described [32].

### Ontology-based Pattern Identification Clustering

3059 genes identified as differentially expressed across the life cycle stages sampled (fold change ≥ 1.5, one-way *p*ANOVA < 0.2) were analyzed as previously described using OPI resulting in 381 gene clusters [32]. Furthermore, a new custom annotated sporozoite-specific GO group (GO:GNF0006) was also included in this analysis resulting in a 382nd OPI cluster 37 genes in size [see Additional file 1]. From these total 382 clusters ranging in size from 37 to 398 genes, 21 were selected on the basis of biological interest for subsequent GEMS analysis.

### Identification of Differentially Expressed Heat Shock Genes

Non-parametric Mack-Skillings test previously described [59] was carried out at the probe level to compare the control and heat shock treated sample. All genes were ranked based on the *p*-value and the top 75 genes (*p *<1.5 × 10^-4^) were defined as the positive set while the remaining 4744 genes were considered the negative set [see Additional file 5].

### Gene Enrichment Motif Searching Analysis

For GEMS analysis of each OPI co-expression cluster, genes within a given cluster are designated as belonging to the positive set while genes outside the cluster (i.e. the remainder of differentially expressed genes) belong to the negative set. Assuming a set of *N *genes in total (positive and negative sets) and a subset of *n *among them determined to share a co-expression pattern (positive set), GEMS expects a motif responsible for gene co-expression (true positive) to be more abundant in *n *promoter or intron sequences than in *N*-*n *promoter or intron sequences, while a motif not responsible for co-expression will distribute more or less randomly among all the *N *promoter or intron sequences. If a motif, represented by a PWM, matches *M *promoter or intron sequences in total and among them *m *genes fall in the cluster of interest, the motif enrichment score is measured by using the hypergeometric distribution to calculate the probability of finding at least *m *matches if one randomly selects *M *genes out of the total *N *gene collection. Therefore, the null hypothesis is calculated as:

$$P(N,n,M,m)=\sum_{i=m}^{min(n,M)}\frac{\left(\begin{array}{c} n\\ i \end{array}\right)\left(\begin{array}{c} N-n\\ M-i \end{array}\right)}{\left(\begin{array}{c} N\\ M \end{array}\right)}$$

The smaller the *p*-value score for a motif, the higher the likelihood the motif explains the observed co-expression of genes included in a given cluster.

In this study, the upstream regions were defined as 1000 base pairs relative to gene start codons. Therefore, in promoter search instances, all sequences were of the same length and *p*-value scores were based on gene counts. However, in the introns searches, to accommodate for the variable lengths of introns in each gene, *N, n*, *M *and *m *in equation 1 were altered to represent base pair counts instead of gene counts. Intron sequences (genes with exons masked out) were identified from the latest genome annotations from PlasmoDB (Release 5.4) [60].

If each motif of a given size and its locations relative to each gene in the data set is pre-calculated and cached in computer memory, the above *p*-value score calculation is greatly accelerated and GEMS becomes feasible on latest computational hardware. GEMS analysis can then be applied to each cluster as follows:

1 For each motif size *l *in (*l*_1_, *l*_2_, ..., *l*_*n*_)

2 *Ω*_*p *_← {all unique words of length *l *appears in either positive or negative sequence sets}.

3 *Ω *← {all unique words of length *l *appears in both positive and negative sequence sets}, i.e. *Ω *= *Ω*_*p *_∪ *Ω*_*n*_

4 Every word *ω *in *Ω *is assigned a score *P*_*ω *_(According to Equation 1)

5 For each word *ω*_*i *_in *Ω*_*p*_

6 An OPI-like parameter optimization process (According to Equation 2)

7 **M**_*i*_(*ω*_*i*_, ε*) is the optimal PWM seeded by *ω*i, where ε* is the optimal mismatches allowed

Using the above hypergeometric-based scoring approach, GEMS begins by scoring and then ranking all *l*-size words (5–8 base pairs for this study) that occur in the positive promoter set *Ω*_*p *_(line 4). The size of *Ω*_*p *_is typically much smaller than the space of all possible *l*-size words (4^*l*^) therefore avoiding a great amount of unnecessary calculations. A PWM is then constructed as a neighborhood in the sequence space that is approximately centered on a given seed word *ω*_*i *_within a vicinity of *ε*. This takes all the words in *Ω*(including those that occurs only in the negative set) that contain no more than *ε *mismatches to *ω*_*i*_. A PWM **M**_*i *_based on the nucleotide frequency in each base position is then constructed with each word weighted by their individual *p*-value score represented as |log_10_*P*|. To avoid assigning subjective parameters for mismatch rate *ε *and similarity threshold *s*, an OPI-like parameter optimization routine is applied to locate the parameters ε * and s* that result in the lowest *p*-value for a given PWM **M**_*i*_. This OPI routine (line 6) can be mathematically summarized as:

$$\begin{array}{c} \left\{\varepsilon^{*},s^{*}\}=\underset{\varepsilon ,s}{\arg\min}P(M_{i}(\omega_{i},\varepsilon ),s\right) \end{array}$$

This minimization routine eliminates the need for subjective selection of parameters typically used in other studies. Thus, the above motif searching routine exhaustively identifies an optimal motif candidate starting from each unique word. To accelerate the calculation, the code is parallelized and run on an 80 node Linux cluster. Heuristics are also taken to prevent repeatedly exploring heavily overlapping neighborhoods. In practice, our CPUs start sequentially from the best-scoring word seeds from each cluster, since good seeds tend to lead to PWMs with better *p*-value scores. Once a PWM is converged, all of the remaining unprocessed words are compared against the newly constructed PWM and unprocessed word seeds that fall into the PWM neighborhood based on s* are excluded from the queue to avoid wasting computational resources. This greedy searching rule drastically reduced the search space in our study.

### Gene Enrichment Motif Searching Motif Clustering

Like most other motif finding algorithms, a PWM discovered can be a sub/super motif of or overlap significantly with another PWM candidate. To address this issue we clustered all motif candidates obtained from a gene cluster based on their locations in a set of promoter sequences using Tanimoto distance [27,61]. This approach was used because simply merging to similar PWMs without position information might have accidentally merged unique PWMs with somewhat similar matrix elements, yet very distinct genome locations. If **M**_*i *_maps to a set of promoter locations {*Z*_*i*_} and **M**_*j *_maps to {*Z*_*j*_} the distance *d*_*ij *_between two motifs *i *and *j *based on Tanimoto distance [62] is:

*d*_*ij *_= 1 - #{ *Z*_*i *_∩ *Z*_*j *_}/#{*Z*_*i *_∪ *Z*_*j*_}

where #{ *Z*_*i *_∩ *Z*_*j *_} refers to the number of unique genome locations shared by both motifs and #{*Z*_*i *_∪ *Z*_*j*_} refers to the number of unique genome locations match either of the two motifs, *d*_*ij *_resulting in a number between 0 and 1. A shared genome location for two motifs was defined as 40% overlap in their bases [63]. For this study, two motifs with a Tanimoto distance less than 0.2 were considered to represent the same motif and the PWM with the better *p*-value score was retained.

### Additional Supporting Evidence

For each gene cluster, the top 20 highest scoring promoter-derived motifs according to *p*-value score were reported [33] and up to ten intron-derived motifs were reported [34]. Also, included in these databases were metrics to aid in the biological assessment of each motif as a putative regulatory element. For example, in the promoter-derived motif database, histograms displaying the frequency of motif locations upstream of gene start codons (100 base pair bins) were provided with red bars representing the distribution of motifs ahead of genes in the positive set and green bars represent the distribution of motifs ahead of genes in the negative set. Enrichment for motif location at particular base pair positions can be considered independent supportive evidence for the biological relevance of a putative regulatory element [25]. Orthologous support from *P. berghei*, *P. y. yoelii*, and *P. chabaudi *was also obtained as independent biological support for each motif. Orthologous *p*-value scores were obtained by projecting *N *and *n P. falciparum *genes to each related species via the ortholog mapping thus obtaining *N*' and *n*' orthologs, respectively. Promoter regions of rodent malaria species were scanned for motif binding sites using the same PWM derived from the appropriate *P. falciparum *sequence region. Assuming *M*' and *m*' motif matches are found within the *N*' and *n*' gene sets, the motif enrichment score in the related species therefore is represented similar to Equation 1:

*P*_ortholog _= *P*(*N*', *n*', *M*', *m*')

The similarity threshold for each related species was individually optimized without modifying the PWM in order to collect the best possible orthologous evidence.

### Motif Summary Analysis

To further reduce the list of 420 promoter-derived motifs produced by GEMS analysis into a short list of highest confidence motifs for future biological validation, we focused on motifs having an orthologous gene log_10_*P *= -2.0 in at least one rodent malaria species resulting in 50 motifs. A CompareACE-like [25] similarity metric with local cluster structures having a minimum internal similarity score of 0.8 was then used to further cluster similar motifs from all gene clusters resulting in the final list of 34 regulatory element candidate groups [see Additional file 3]. In regards to intron-derived motifs, we focused on those having *P. falciparum *enrichment scores log_10_*P *= -3.0 and orthologous gene enrichment scores log_10_*P *= -2.0 in at least one rodent malaria species, resulting in 32 motifs. A compareACE-like [25] similarity metric with local cluster structures having a minimum internal similarity score of 0.8 was then used to further cluster similar intron-derived motifs from all gene clusters resulting in the final list of 26 regulatory element candidate groups [see Additional file 4]. The same similarity metric was also applied to associate intron-derived motifs with promoter-derived motifs for the convenience of analysis.

### MDScan and MEME analysis

For the MDScan analysis, the latest available version of MDScan as of July 2007 was downloaded [64]. The positive set sequences were ranked based on their expression similarities to the query expression pattern of the Sexual Development OPI cluster (GO:GNF0004), (i.e., the genes closer to the top are more likely to be true positives, therefore in agreement with the design principle of MDScan). Promoter sequences were optionally preprocessed using RepeatMasker [65], where a 100 base pair stretch of DNA was masked if it was >87% AT or >89% GC, and a 30 base pair stretch was masked if it contained >29 A/T or G/C nucleotides. According to the recommendation of MDScan documents, single and dinucleotide repeat sequence fragments of six bases or longer were always masked out to avoid convergence on such non-sense motifs [64]. Background sequences were either directly supplied to MDScan program using -b option or as a model trained by genomebg.linux program. With all the above variations of input sequences and parameters, a total of 16 MDScan runs were initiated for motif length of 5 to 8 bases. The top ten best-scoring motif candidates ranked by MAP score were retained. From the output file, we collected PWMs and all the matched words, then identified all the genes that contained such words. A cluster-specificity score was then calculated using accumulative hypergeometric *p*-value as recommended by previous studies [25]. To further study whether the MAP scores were significantly tied to a given gene cluster, we also carried out a full permutation simulation, where the same number of positive sequences were randomly selected and the above whole analysis process were repeated 100 times.

For the MEME analysis, the latest available version of MEME as of July 2007 was used (v.3.5.4). The promoter sequence files used were the same as those used in the MDScan analysis. Sequences were optionally preprocessed using RepeatMasker as described for the MDScan analysis. Background sequences were used to train 0th, 1st and 2nd order background Markov models. MEME runs were also initiated using zoops or anr for its -mod option. A total of 12 MEME runs were initiated for motif length of 8 bases. The top ten best-scoring motif candidates were retained, whenever possible. MEME motif candidates were retrieved in the form of PWMs and the MAST program was applied to identify all transcription factor binding sites in both positive and background sequence sets. A cluster-specificity score was then calculated using accumulative hypergeometric *p*-value as recommended by previous studies [25].

### Construction of pPf-RAP3-86 and pPf-RAP3-Del-86

The 1527 base pairs upstream of the *rap3 *(PFE0075c) start site were PCR amplified from *P. falciparum *strain 3D7 genomic DNA using primers RAP3-5UTR-F (GATCGACTCGAGGACATTTAAATATTATATTACAAGGAAAAGGAC) and RAP3-5UTR-R (CTGATCCCATGGTTTAAAAGTCTTAAATATTATATTAATAAATTTATAAAAC). The primers contained 5' XhoI and NcoI restriction digestion sites for downstream cloning. The PCR product was ligated into the pGEM-T-Easy TA-cloning vector (Promega) to create pGEM-RAP3-5UTR. The 16 base pair motif was deleted using overlap extension PCR by first amplifying the 3' 641 base pairs of the RAP3 5' UTR using primers RAP3-5UTR-del-F (TAAAAAAAAAGGAAAAGAAAATTAAAGTATTATTTTTAAATAAAAAATAAAATAAAATAAACTCTCTTTTGAATGAAT) and RAP3-5UTR-R. The RAP3-5UTR-del-F primer flanks the 16 base pair motif on both sides to amplify the surrounding regions while deleting the motif. The resulting fragment was used with RAP3-5UTR-F to prime the amplification of the remainder of the RAP3 5' UTR. This product was ligated into the pGEM-T-Easy vector to create pGEM-RAP3-5UTR-Del.

The pPf86 vector, which contains the firefly luciferase gene flanked by the Hsp86 5' and 3' UTRs, was obtained from Prof. Dyann Wirth's laboratory [66]. pPf86, pGEM-RAP3-5UTR and pGEM-RAP3-5UTR-Del were digested with XhoI and NcoI, the Hsp86 5' UTR was excised from pPf86 by digestion with XhoI and NcoI and replaced with both the full and deleted versions of the RAP3 5' UTR to create pPf-RAP3-86 and pPf-RAP3-Del-86, respectively.

### Parasite Transfection

Blood-stage *P. falciparum *strain 3D7 parasites were cultivated and synchronized using standard procedures [56,67]. Parasites were transfected with either pPf-RAP3-86 or pPf-RAP3-Del-86 by invasion of DNA-loaded red blood cells [68]. Briefly, erythrocytes were cleared of leukocytes, washed with RPMI 1640 (Invitrogen) and resuspended to 50% hematocrit. 350 μl aliquots of erythrocytes were washed with 5 ml of incomplete cytomix [69] and resuspended in cytomix containing 50 μg of DNA to a total volume of 400 ul. The aliquots were transferred to a 0.2 cm cuvette and chilled on ice. Cells were electroporated with a Bio-Rad Gene Pulser using 0.31 kV and 960 μF. Each 25 cm^2 ^cell culture flask (Corning) containing 2 aliquots of DNA-loaded red blood cells in 9 mL of cell culture medium was inoculated with 1 mL of trophozoite-stage synchronized parasite culture at approximately 5% parasitemia (approximately 2.5 × 10^7 ^parasites). In total, 24 flasks were prepared such that 2 flasks were harvested for each of 6 time points and for each of the 2 vectors transfected.

### RNA Purification and Real Time PCR

Parasites were harvested and RNA purified every 8 hours over a 48-hour time period using Trizol according to manufacturer's instructions (Invitrogen), with care being taken to extract the same volume from the aqueous phase of each sample. The first time point was taken at the early ring stage of the parasite life cycle. Total RNA was treated with Dnase I (New England Biolabs). The RNA was reversed transcribed from total RNA using random primers and Superscript III (Invitrogen), after which RNA was degraded with RNase H (Invitrogen). The luciferase mRNA was amplified and quantified from random-primed cDNA template using iQ supermix (Bio-Rad) and primers Luc-RT-F (GCGAACTGTGTGTGAGAGGTCCTATG) and Luc-RT-R (TTACACGGCGATCTTTCCGCCCTTC) on a Bio-Rad iCycler PCR machine with MyiQ real-time detection module. As a control, the *P. falciparum *β-tubulin mRNA was also quantified using primers B-Tub-RT-F (TCGTCAACTTCCTTTGTGGA) and B-Tub-RT-R (TCCCATTCCCACGTTTACAT).

### Electrophoretic Mobility Shift Assays

Mixed asexual stage NF54 parasites were obtained from a ~9.0% parasitemia culture using saponin lysis. Nuclear extracts were isolated from the resulting parasite pellets using NE-PER reagents according to manufacturer instructions (Pierce). Nuclear extracts were then immediately flash frozen in liquid N_2 _and stored at -80°C until needed.

30 – 34 mer oligos containing motif sequences of interest or random sequences for competition experiments were obtained as reverse complements and in PAGE purified form (Integrated DNA Technologies). [O197-TCGATATTTTTATGCATGTGAAGTGCAAAATAAA. O198-TTTATTTTGCACTTCACATGCATAAAAATA. Forward 80AT-GATCTAATCAGTATTCAAGAGTACTTATTAAATT. Reverse 80AT-AATTTAATAAGTACTCTTGAATACTGATTA. Forward 20AT-GATCGGCCGGCCGCCAAGGTCCCGTGATGCGCGC. Reverse 20AT-GCGCGCATCACGGGACCTTGGCGGCCGGCC.] In the case of radiolabeled probes, oligonucleotide pairs were annealed using a step down protocol in a thermocycler (95°C 5 min, 95°C 1 min, -1°C/cycle for 69 cycles, 4°C 10 min) and the resulting four base pair overhangs were labeled by incubating 200 pmoles of annealed oligos with 50 units Klenow enzyme (New England Biolabs) and dCTP-α^32^P (20 μCi), dATP, dGTP and dTTP (50 mM final concentration each) for 120 min at 37°C. These reaction mixtures were then cleaned up with free unincorporated nucleotides being removed using size exclusion G-25 columns (Amersham). In the generation of cold competitor probes, all steps were the same with the exception of dCTP-α^32^P being replaced with non-radioactive dCTP.

For each EMSA reaction, nuclear extracts (~6.4 μg) were incubated in ×1 EMSA buffer (20 mM HEPES pH 7.8, 1 mM DTT, 100 mM KCl, 0.5 mM EDTA, 1 mM MgCl_2_, 0.5 mM ZnCl_2_, 50 ng/μL Poly(dIdC)) for 20 minutes at room temperature. If applicable, cold competitor probe (4 or 20 pmol) was also included in this pre-incubation step. Radiolabeled probe (1 pmol) was then added to the reaction mix bringing the total reaction volume to 20 μL and the mix was incubated for 5 min at room temperature. The binding reactions were analyzed on a 5% polyacrylamide gel in 0.5× TBE and quantified using a phosphoimager system.

## List of Abbreviations

GEMS: Gene Enrichment Motif Searching; PWM: Position-Weight Matrix; EMSA: Electrophoretic Mobility Shift Assay; OPI: Ontology-based Pattern Identification; GO: Gene Ontology; RAP: Rhoptry-Associated Protein; TCA: Tricarboxylic Acid; MOID: Match-Only Integral Distribution.

## Authors' contributions

JAY carried out microarray and molecular studies, as well as drafted the manuscript. JRJ carried out molecular studies. KGL carried out microarray studies. CB, SFY, and KC conducted statistical and bioinformatics analyses. YZ and EAW conceived of the study, participated in its design, conducted bioinformatics analyses, and helped draft the manuscript. All authors read and approved the final manuscript.

## Supplementary Material

Additional File 1 Sporozoite-specific OPI cluster seed genes. The 18 *P. falciparum *sporozoite-related genes used to seed OPI generation of the sporozoite-specific gene cluster GO:GNF0006. Click here for file

Additional File 2 OPI clusters used for GEMS analysis. The 21 OPI clusters used as the basis for GEMS analysis listed by GO identifier number and cluster size. Click here for file

Additional File 3 Putative promoter *cis*-regulatory elements discovered by GEMS. Motif derived from GEMS analysis of 1000 base pair promoter regions upstream of gene start codons. Click here for file

Additional File 4 Putative intron *cis*-regulatory elements discovered by GEMS. Motif candidates derived from GEMS analysis of gene intron regions. Click here for file

Additional File 5 Differentially expressed genes in response to heat shock. Top 75 differentially expressed genes in response to heat shock treatment identified by microarray hybridization and non-parametric Mack-Skillings test. Click here for file

Additional File 6 *P. falciparum *mitochondrial TCA cycle gene expression. Life cycle mRNA expression values for *P. falciparum *mitochondrial TCA cycle genes as determined by microarray hybridization. Click here for file
